# Comparison of Models Fitting Alternative Contemporary Group Definition and Data Editing Procedures in the Genetic Evaluations of Reproductive Performance and Cow Longevity Traits in Nellore Cattle

**DOI:** 10.3390/ani16203222

**Published:** 2026-10-13

**Authors:** Andreia Kaspary Zwirtes, Daniele Alves de Oliveira, Saymon Silva Rocha, Delvan Alves Silva, Luiz Fernando Brito, Larissa Bordin Temp, Nathalia Pereira Seixas, Roberto Carvalheiro, Lucia Galvão de Albuquerque, Thaise Pinto de Melo

**Affiliations:** 1 Animal Science Department, Federal University of Santa Maria, Santa Maria 97105-900, RS, Brazil; andreiakasparyzoot@gmail.com (A.K.Z.); daniele.alves@acad.ufsm.br (D.A.d.O.); saymon.rocha@acad.ufsm.br (S.S.R.); nathaliaaseixasp@gmail.com (N.P.S.); 2 Department of Animal Science, Federal University of Viçosa (UFV), Viçosa 36570-900, MG, Brazil; delvan.silva@ufv.br; 3 Department of Animal Sciences, Purdue University, West Lafayette, IN 47907, USA; britol@purdue.edu; 4 Department of Animal Science, São Paulo State University (UNESP), Jaboticabal 14884-900, SP, Brazil; larissabordintemp@gmail.com (L.B.T.); galvao.albuquerque@unesp.br (L.G.d.A.); 5 Commonwealth Scientific and Industrial Research Organisation (CSIRO), Hobart, TAS 7000, Australia; roberto.carvalheiro@unesp.br

**Keywords:** accuracy, bias, dispersion, heritability, reproductive traits

## Abstract

Reproductive traits in beef cattle are strongly influenced by environmental and management factors, making the correct definition and quality control of contemporary groups (CG) a key step for reliable genetic evaluations. Therefore, the main objective of this study was to evaluate the impact of alternative models considering different definitions of CG, minimum CG sizes (MCGS), and standard deviation thresholds for phenotypic outlier removal on the genetic evaluation of cow reproductive and longevity traits in Nellore cattle (*Bos taurus indicus*). The four traits evaluated included age at first calving (AFC; in days), early pregnancy occurrence (EP; binary), scrotal circumference at 18 months (SC18; in cm), and total number of calvings at 53 months of age (NC53; count). The database comprised 954,216 animals, including 401,885 females and 552,331 males, with 227,447, 401,885, 356,519, and 531,579 records available for AFC, EP, NC53, and SC18, respectively, and a pedigree file comprising 1,348,164 animals. Variance components and genetic parameters were estimated using a pedigree-based animal model implemented in the BLUPF90 software family (version 1.57) for continuous traits (AFC and SC18) and a Bayesian animal model implemented in THRGIBBS1F90 (version 2.108) for categorical traits (EP and NC53). The Linear Regression (LR) method was used to assess model performance based on accuracy, bias, and dispersion of estimated breeding values (EBVs). The main CG criteria included MCGS of 3, 7, and 15 animals, four phenotypic outlier-removal thresholds (±2.0, ±2.5, ±3.0, and ±3.5 SD) for continuous traits, and the presence or absence of phenotypic variability within CGs for categorical traits. We also compared alternative combinations of fixed effects included within or outside CGs and evaluated genetic connectedness among CGs. The definition of CGs and data editing procedures yielded different heritability estimates and EBV accuracies across scenarios and traits. The heritability estimates for the evaluated traits ranged from 0.06 to 0.45, while EBV accuracies ranged from 0.45 to 0.92 across CG scenarios. Continuously-recorded traits, especially AFC, were more sensitive to model specification and CG structure, whereas categorical traits and SC18 were more robust to differences in quality control criteria. Animal rankings showed high consistency among scenarios, with mean concordance of 94.8% for the top 1% of males and top 20% of females and 95.4% for all animals. Our findings indicate that the definition of CGs and data editing procedures for each trait and population are key steps to be carefully performed before genetic evaluation analyses, since parameters such as heritability estimates, EBV accuracy, bias, and dispersion may be affected.

## Simple Summary

Genetically improving reproductive performance and productive life in beef cattle is essential for increasing the efficiency and sustainability of the sector. However, these traits are largely affected by environmental factors, making it difficult to accurately identify animals with the best genetic potential for them. This study aimed to evaluate different strategies for grouping animals that experienced similar management and environmental conditions when estimating their genetic merit. Four traits related to cow reproductive performance and longevity in Nellore cattle were analyzed using records from more than 800,000 animals. The results indicate that the definition of contemporary groups can influence the estimation of breeding values for the traits evaluated in this study, especially for age at first calving, while other traits were less affected. Despite these differences, the ranking of the best animals remained highly consistent across the model definitions. The careful consideration of environmental effects is more important than simply increasing the number of animals in each group through broader definitions of contemporary groups and data editing strategies. These findings provide practical guidance for breeding programs to improve the accuracy of genetic evaluations, thereby enabling more effective selection of genetically superior animals in beef cattle populations. In the long term, this contributes to healthier, more productive, and longer-living cattle, improving the sustainability and profitability of beef production.

## 1. Introduction

Reproductive performance traits are key indicators of profitability in beef cattle production systems [1]. Zebu cattle breeds (*Bos taurus indicus*) generally grow and mature sexually more slowly than taurine breeds (*Bos taurus taurus*) [2]. In this regard, genetic selection can be an effective tool for increasing reproductive efficiency in beef herds [3]. Over the past few decades, beef cattle breeding programs have prioritized reproductive performance traits in selection schemes, including age at first calving, early pregnancy occurrence, and scrotal circumference at 18 months as an indicator of heifer precocity [4,5].

Traits such as sexual precocity and cow longevity generally have low heritability estimates due to the high influence of environmental and management factors and they are also highly polygenic and influenced by many genes with small effect on the traits phenotypic expression [6]. In this context, the proper adjustments of phenotypic records for environmental factors in genetic evaluation models can contribute to reducing the residual variances (errors) from the models and potentially improve the estimates obtained.

Contemporary groups (CG), defined as sets of animals exposed to similar environmental and management conditions, are commonly fitted as fixed effects in genetic evaluations to account for systematic environmental differences among groups [7]. A recent study by Carreño et al. [8] showed that group size and the inclusion of contemporary groups as fixed effects can influence estimates of genetic parameters, highlighting the importance of appropriately defining group structure in genetic evaluation models. The definitions of CG vary according to the species, breed (or line), production and management systems, dataset size and structure, and traits evaluated. In addition to the effects included in the definition of CG, other factors may also influence the choice of the best genetic evaluation model, such as the minimum number of animals per CG [9,10] or the number of standard deviations from the CG mean considered for the removal of outliers for continuously-recorded traits that follow a Gaussian distribution.

Despite multiple definitions of CG being used in genetic evaluation models for reproductive traits in beef cattle, there is no consensus on quality control criteria for CG, such as minimum CG sizes (MCGS), phenotypic variation within groups, and concatenated effects within CG [8]. Nevertheless, the strategies used to define CG can affect the estimation of (co)variance components and genetic parameters, the connectedness between CG, and the accuracy of EBVs [11,12]. Inadequate definition of CG can lead to incorrect selection and culling decisions, resulting in overestimated or underestimated predictions of EBVs [13].

Although genomic information can improve the estimation of genetic relationships and model fit, pedigree-based animal models remain relevant for evaluating genetic parameters and population structure, particularly when the objective is to investigate the effects of model assumptions and contemporary group definition. Recent evidence from composite beef cattle indicates that incorporating genomic information can improve model fit and modify variance component estimates [13,14].

Despite the importance of CG definition and quality control for genetic evaluations, there is still no consensus on the most appropriate criteria for defining and filtering CG data, particularly for reproductive traits in *Bos taurus indicus* populations. Previous studies have focused on specific aspects of CG structure or individual quality-control criteria, whereas the consequences of alternative scenarios for the overall quality of genetic evaluations remain unclear. Thus, the main objective of this study was to evaluate the impact of different definitions of CG quality-control criteria on genetic parameter estimates, accuracy, bias, dispersion, connectedness between CG, and animal classification in the genetic evaluation of age at first calving (AFC), scrotal circumference at 18 months (SC18), total number of calving at 53 months of age (NC53), and early pregnancy occurrence (EP) in Nellore cattle (*Bos taurus indicus*).

## 2. Materials and Methods

### 2.1. Study Design

The data used in this study were obtained from the Aliança Nelore breeding program (Gensys—Porto Alegre, RS, Brazil) and comprised records of animals raised on pasture in different commercial herds located in the Midwest and Southeast regions of Brazil and in Paraguay. The database contained 954,216 animals, including 401,885 females and 552,331 males, and the pedigree file included 1,348,164 animals. The traits evaluated included AFC, defined as the number of days from birth to first calving, EP, which was classified as success (1) for heifers that calved by 31 months of age, i.e., heifers that became pregnant in the first breeding season, or failure (0) for heifers whose AFC was greater than 31 months of age; NC53, which ranged from 0 to 3 calvings. The comparison was made between females that calved up to 3 times at 53 months of age and those that calved 1, 2, or those that never became pregnant (0); and SC18, which was measured in centimeters around 18 months of age at the widest point of the scrotum with a standard tape measure. A total of 227,447, 401,885, 356,519, and 531,579 records were available for AFC, EP, NC53, and SC18, respectively.

The mean AFC and SC18 were 1014 ± 130.27 days and 26.67 ± 3.77 cm, respectively. For the categorical traits, 82.7% of the animals were classified as EP = 0, whereas 17.3% were classified as EP = 1. For NC53, 46.78% of the animals were grouped in class 0, 22.97% in class 1, 29.15% in class 2, and 1.10% in class 3.

Heifers were naturally mated or artificially inseminated during the rainy season, which generally occurred between October and March. In general, females were exposed for the first time at around 26 months of age, but some herds started the breeding process earlier, between 14 and 18 months of age. Those heifers that did not conceive in the first breeding season at an early age had a second chance at 26 months during the regular breeding season. Heifers that did not conceive on either breeding season were culled.

The months of birth were grouped into two seasons to level out the environmental effects related to different climatic conditions throughout the year: season 1 (rainy season; October to March) and season 2 (dry season; April to September). The age of the cows at calving was used as a covariate in linear and quadratic forms, with an average of 5.83 years (±2.94 years). For SC18, the age at the measurement was also included in the model as a covariate (linear and quadratic effects).

### 2.2. Model Description for CG

The impact of three data editing criteria on the definition of CG was evaluated. The first was the minimum number of animals per CG, i.e., 3, 7, and 15 animals, as proposed by Silva et al. [8]. These MCGS represent progressively more restrictive levels of data editing and enabled the evaluation of the trade-offs between retaining a more representative number of animals in the analyses and improving the statistical robustness of the CG effect estimates. The second criteria involved variability within CGs, which was defined differently for: (i) continuously-recorded traits (AFC and SC18), with four standard deviation (SD) thresholds (±2.0, ±2.5, ±3.0, and ±3.5) from the CG mean for removing phenotypic outliers, totaling twelve analyses with different combinations of SD thresholds and MCGS used; and (ii) categorical traits (EP and NC53), in which data exclusion was based on the presence or absence of variability within the CGs (groups with two or more categories for the response variable vs. groups with only one category), totaling six analyses with different combinations of MCGS. The third criterion involved different definitions of CG based on alternative combinations of significant fixed effects included within or outside the CG, resulting in eight models for AFC, five models for EP, four models for NC53, and seven models for SC18.

For AFC and SC18, 12 scenarios with different data structures (E1–E12) were evaluated, combining criterion 1 and 2, MCGS, and different phenotypic outlier removal thresholds. The outlier removal thresholds evaluated ranged from ±2.0 to ±3.5 standard deviations (SD) from the corresponding CG mean. These thresholds were considered as alternative sensitivity scenarios to evaluate the effect of different levels of phenotypic outlier removal on the genetic evaluation results. The MCGS was set at 3, 7, or 15 animals. Thus, scenarios E1 to E3 considered a threshold of ±2.0 SD; E4 to E6 ±2.5 SD; E7 to E9 ±3.0 SD; and E10 to E12 ±3.5 SD, each combined with the respective MCGS of 3, 7, and 15 animals per group.

Similarly, for EP and NC53, six scenarios (E1–E6) were tested, resulting from the combination of the presence or absence of variability within the CG and the minimum number of animals per CG (3, 7, and 15). Structures E1, E3, and E5 considered the presence of variability within CGs, while E2, E4, and E6 were defined allowing the absence of variability within CG (only a categorical response). This approach enables us to evaluate the simultaneous impact of the level of quality control on CG connectedness and the other genetic parameters evaluated.

To evaluate different CG definitions and consequently construct the alternative models, the significance of each candidate fixed effect was first evaluated using the SAS program through the PROC GLM procedure (v. 9.4, SAS Institute Inc.; Cary, NC, USA). Significant effects (*p* < 0.001) associated with birth cohort, birth season, farm, management groups at different production stages, cow age at calving (linear and quadratic effects), and male age at yearling (linear and quadratic effects) were retained for the construction of the different models across traits.

The effects tested were: birth cohort (from 1984 to 2019), birth season (1: spring and summer; season 2: fall and winter), birth farm (525 farms), management group from birth to weaning of the first calf (W1OMG; *n* = 7201) management group from birth to weaning of the animal (WMG; *n* = 6999), animal yearling management group (YMG; *n* = 6788); LAC: linear effect of cow age at calving; QAC: quadratic effect of cow age at calving, LAY: linear effect of yearling age of males on SC18 assessment; QAY: quadratic effect of yearling age of males on SC18 assessment.

**Figure 1:**
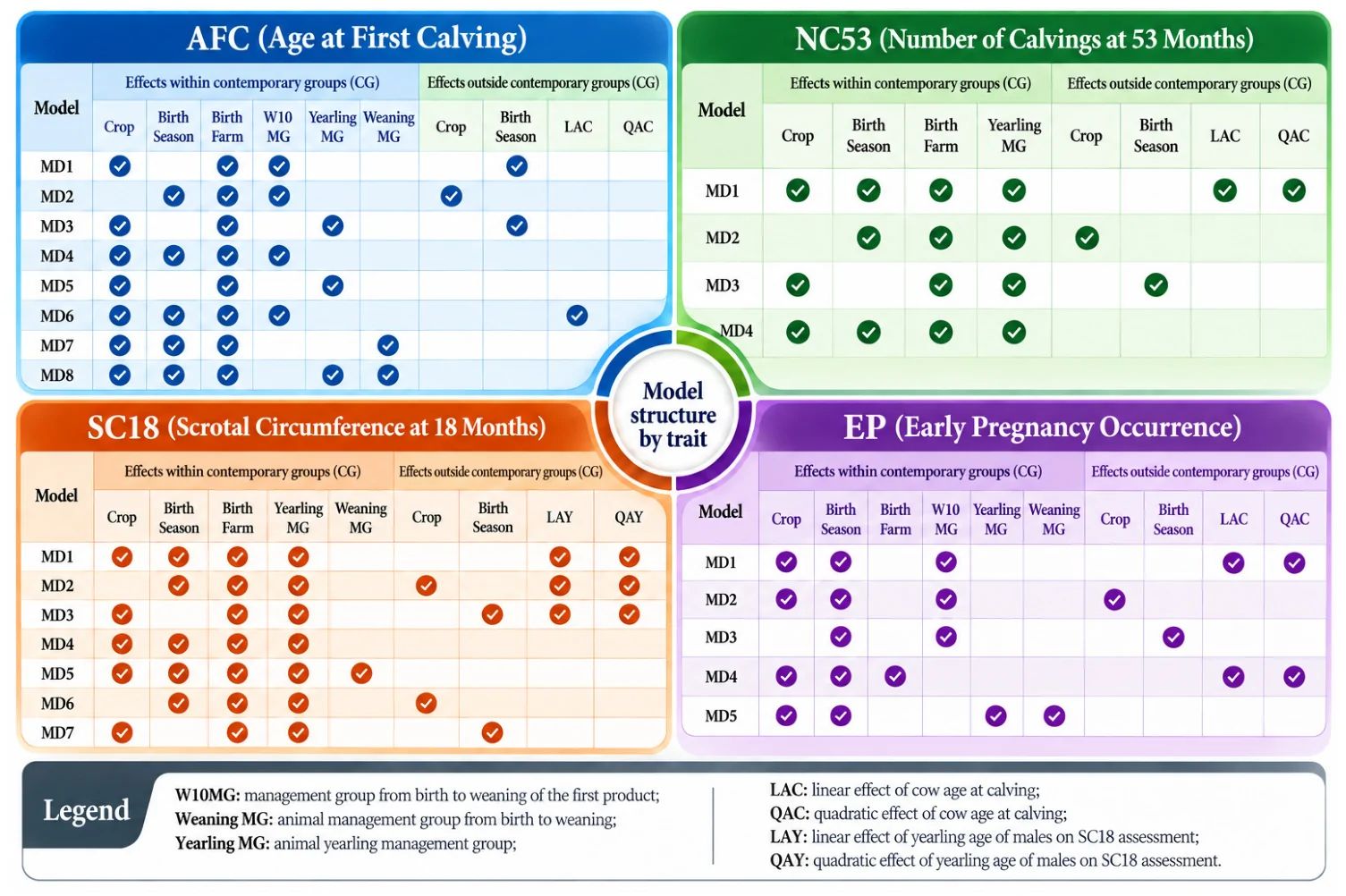
Structure of the models (MD) evaluated for age at first calving (AFC, MD1–8), early pregnancy (EP, MD1–5), total number of calvings until 53 months of age (NC53, MD1–4), and scrotal circumference measured at 18 months of age (SC18, MD1–7) in Nellore cattle, varying according to the inclusion or exclusion of fixed effects. A check mark indicates the fixed effects included in the models. MDi: evaluated models, where *i* represents the model number for each trait.

Figure 1 shows the different models evaluated for AFC, EP, NC53, and SC18. The fields marked in each table indicate whether the effects were included within (concatenated) or outside (independent fixed effects) the CG. This strategy was adopted for some of the fixed effects included in the model in order to assess whether there was an impact on concatenating the effect within CG or evaluating it independently. The descriptive statistics of the models evaluated for the different MCGS are presented in Supplementary Tables S1–S4.

The following parameters were used to evaluate the impact of the different criteria (minimum numbers of animals, SD limits for phenotypic outlier removal, and CG definitions): (I) estimates of variance and heritability, (II) EBV prediction accuracy, (III) EBV bias, IV) EBV dispersion, (V) connectedness between CGs, which was evaluated using the AMC software (version 4.1) [15]. Contemporary groups were considered genetically connected when they had at least 10 genetic links with other CGs. Genetic links were defined as direct links between CGs resulting from common sires and/or dams, based on traditional pedigree information. The total number of genetic links was calculated as the sum of the links due to common sires and dams. The threshold of 10 genetic links was adopted as suggested by Roso et al. [16] and (VI) animal ranking (1.0% best males and 20.0% best females based on EBVs for each trait).

For practical comparison, models were considered similar when differences among them were lower than 5.0% for heritability and accuracy estimates and lower than 0.05 units for bias and dispersion statistics across all evaluated MCGS scenarios. These criteria were defined empirically based on the magnitude and variability of the estimates observed across the evaluated scenarios and were intended to identify models with relatively small differences in performance.

Accuracy, bias, and dispersion of EBVs were obtained using the LR (Linear Regression) method proposed by Legarra and Reverter [17]. Accuracy was calculated as the correlation between the EBVs of the validation population (reduced dataset) and EBVs for the same animals calculated based on the full dataset (all animals with phenotypic records). The validation population consisted of younger animals from birth cohorts 2016, 2017, and 2018 for AFC, NC53, and EP, and birth cohorts 2017 and 2018 for SC18. The EBV accuracy was calculated as:

(1) $$\rho_{\left(p,w\right)}=\frac{\mathrm{cov}({û}_{p},{û}_{w})}{\sqrt{\mathrm{var}}({û}_{w})\;\mathrm{var}({û}_{p})},$$

where û*_p_* and û*_w_* are the vectors of EBVs for the validation animals obtained using the reduced and full datasets, respectively.

Bias was estimated as the difference between the means of the EBVs of the animals in the validation population estimated in the full and reduced datasets:

(2) $$\mu_{w,p}=(\underline{{û}_{p}}-\underline{{û}_{w}}),$$

where $\underline{{û}_{p}}$ and $\underline{{û}_{w}}$ are the mean EBVs of the validation animals obtained using the reduced and full datasets, respectively.

Dispersion (*b_w_*_,*p*_) was calculated by regressing the EBVs obtained with the full dataset on the EBVs obtained with the reduced dataset [17]:

(3) $$b_{w,p}=\frac{\mathrm{cov}({û}_{w},{û}_{p})}{\mathrm{var}({û}_{p})},$$

### 2.3. Genetic Evaluation Models

The base model employed for the analysis is shown in Equation (1):

**y** = **Xβ** + **Za** + **e**, (4)

where **y** is the vector of phenotypic observations (AFC, EP, NC53, and SC18), **X** is the incidence matrix relating phenotypic records to systematic effects, **β** is the vector of systematic effects, including the alternative CG definitions, **Z** is the matrix relating animal additive genetic effects to phenotypic records, **a** is the vector containing additive genetic effects, and **e** is the vector containing the residual effects. We considered **a** and **e** to be normally distributed with Var(**a**) = **A**$\sigma_{a}^{2}$ and Var(**e**) = **I**$\sigma_{e}^{2}$, and the covariances between **a** and **e** were assumed to be zero. **A** is the (pedigree-based) additive relationship matrix and **I** is an identity matrix. The analyses were performed using the BLUPF90 program family (version 1.57) [18] for continuously-recorded traits with a frequentist approach (AFC and SC18) and THRGIBBS1F90 (version 2.108) for categorical traits with a Bayesian approach (EP and NC53). For the latter, a total of 1,000,000 chains were run with a burn-in of 3000 and a thin of 100, totaling 9970 chain samples. Convergence was assessed through visual inspection of trace plots, Geweke test, and autocorrelation diagnostics using the BOA R package (version 1.1.9) [19].

## 3. Results

### 3.1. Descriptive Statistics of the Evaluated Scenarios

For the traits AFC and EP, the impact on the number of animals excluded from subsequent analyses was more pronounced when excluding CG by the MCGS criteria. For AFC, the number of phenotypic records ranged from 136,790 (MD7, MCGS = 15) to 201,605 (MD3, MCGS = 3), with an average reduction of population size of 9.48% and 12.34% in MCGS = 15 compared to 7 and 3, respectively, and the number of CG ranged from 2403 (MD3, MCGS = 15) to 12,190 (MD7, MCGS = 3), with a reduction of 29.22% and 41.68% when comparing MCGS = 15 to 7 and 3, respectively (Supplementary Table S1). For EP, the number of phenotypic records ranged from 93,601 (MD4, MCGS = 15) to 312,887 (MD3, MCGS = 3), while the number of CG ranged from 2165 to 8403, with reductions of 21.2% and 29.0%, respectively, when MCGS = 15 was compared with MCGS = 7 and MCGS = 3 (Supplementary Table S2).

NC53 and SC18 also showed reductions when MCGS varied, although to a lesser extent. In NC53, the number of phenotypic records ranged from 347,973 (MD4, MCGS = 15) to 354,626 (MD3, MCGS = 3 and 7), with an average reduction of 2.4% to 2.5% in MCGS = 15 compared to 7 and 3, respectively, and the number of CGs ranged from 2543 (MD3, MCGS = 15) to 4137 (MD1/MD4, MCGS = 3), with a reduction of 11.4% and 12.9% (Supplementary Table S3). For SC18, the number of phenotypic records ranged from 456,082 (MD5, MCGS = 15) to 518,155 (MD3, MCGS = 3), with an average reduction of 2.0% and 2.1%, and the number of CG ranged from 4078 (MD3/MD7, MCGS = 15) to 15,017 (MD5, MCGS = 3), with a reduction of 10.4% and 11.8% (Supplementary Table S4).

### 3.2. Connectedness Among Contemporary Groups

The average number of genetic ties among CG increased with increasing MCGS for all evaluated traits, as expected. For AFC, the average number of genetic ties was 10,825.14, ranging from 4410.91 (MD7, MCGS = 3) to 24,300.45 (MD3, MCGS = 15). MD1 with MCGS = 15 presented the highest connectedness, with 99.43% connected animals and 98.84% connected CG. Detailed connectedness results for all models and MCGS are presented in Supplementary Tables S9–S12 for AFC, EP, NC53, and SC18, respectively.

For EP, the average number of genetic ties was 29,124.56, ranging from 4742.14 (MD5 and MD8, MCGS = 3) to 56,678.44 (MD3, MCGS = 15). The MD3 model showed the highest connectedness level, reaching 99.67% connected animals and 99.06% connected CGs. The exclusion of CGs based on phenotypic variability had little influence on the proportion of connected animals (97.81–98.83%) or CGs (99.30–99.37%). However, it affected the average number of genetic ties. When CGs with low or no phenotypic variability were retained, the average number of genetic ties increased to 39,856.59, whereas excluding these CGs reduced the average number of genetic ties to 24,838.59.

For NC53, connectedness remained highly stable across all evaluated models, with an overall average of 36,069.45 genetic ties. Individual values ranged from 14,120.87 (MD4, MCGS = 15) to 51,912.19 (MD3, MCGS = 15). The MD3 model produced the highest connectedness, with 99.50% connected animals and 98.53% connected CGs under MCGS = 15. Excluding CGs due to low phenotypic variability had little effect on connectedness, with the proportion of connected animals ranging from 99.19% to 99.50% and connected CGs from 97.00% to 98.53% across all scenarios.

For SC18, the average number of genetic ties was 128,895.48, ranging from 51,256.10 (MD5, MCGS = 3) to 185,109.40 (MD3, MCGS = 15). Unlike for the other traits, the SD thresholds used for phenotypic outlier removal directly influenced the number of genetic ties. As expected, more restrictive (lower) SD thresholds excluded a greater number of animals, thereby reducing pedigree links among CGs, whereas less restrictive SD thresholds preserved a larger number of genetic connections. Consequently, the MD5 scenario with MCGS = 3 produced the lowest average number of genetic ties (51,256.10), whereas MD3 with MCGS = 15 produced the highest (185,109.40), representing an increase of approximately 261%. Despite these differences, the proportions of connected animals and CGs remained consistently high across analyses. The MD3 and MD7 models with MCGS = 15 achieved the highest values of connectedness, with 97.77% connected animals and 98.98% connected CGs. Across all scenarios, the proportion of connected animals ranged from 91.46% to 97.77%, whereas the proportion of connected CGs ranged from 94.01% to 98.98%.

### 3.3. Estimates of Variances and Heritability, Accuracy, Bias, and Dispersion

**Figure 2:**
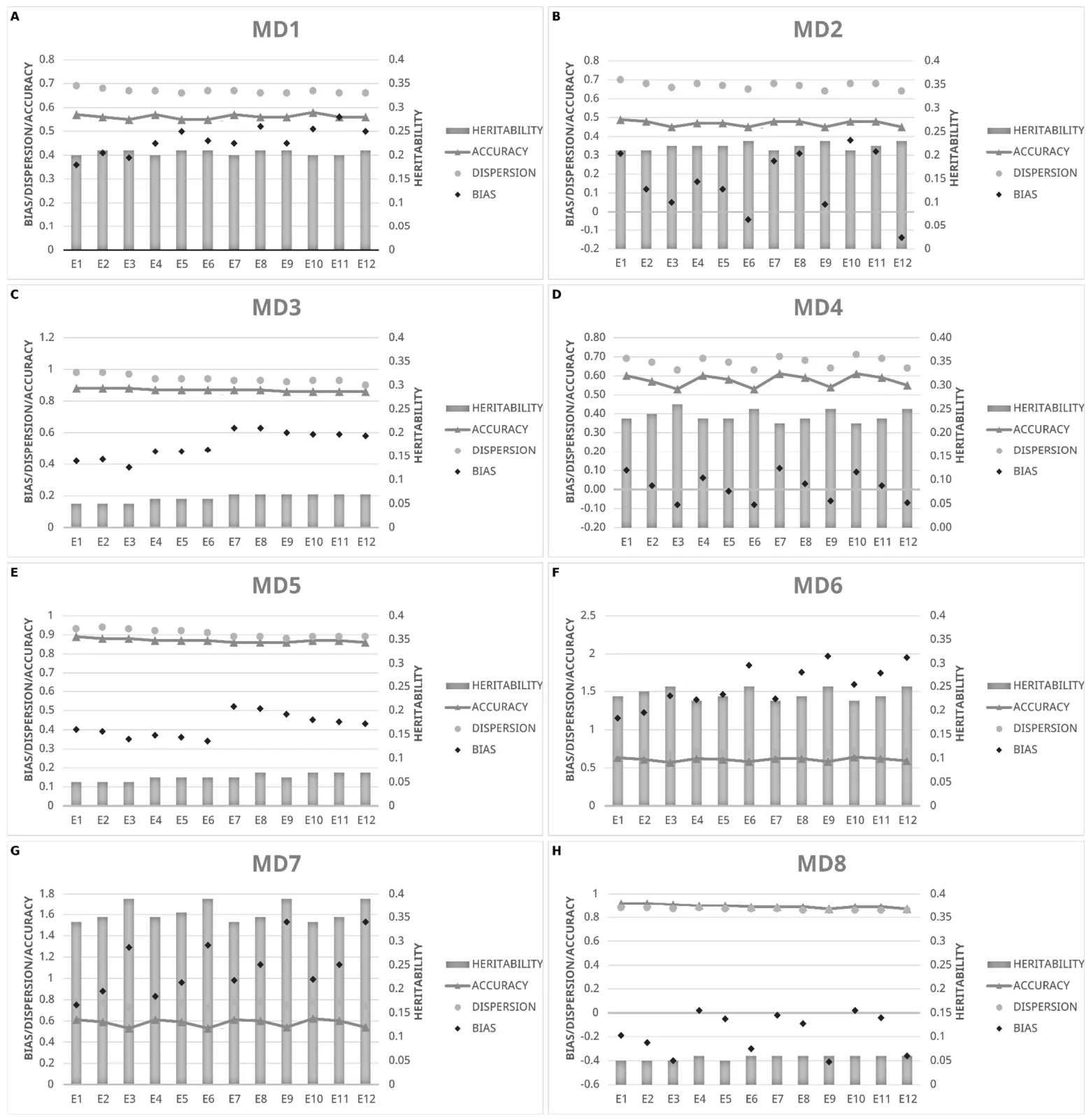
Estimates of heritability, accuracy, bias and dispersion across scenarios (E1–E12) for the MD1–8 models fitted for age at first calving in Nellore cows. Scenarios: E1: minimum of 3 animals per contemporary group (CG) with a SD threshold of 2.0 from the CG mean for removing phenotypic outliers; E2: minimum 7 animals SD 2.0; E3: minimum 15 animals SD 2.0; E4: minimum 3 animals SD 2.5; E5: minimum 7 animals SD 2.5; E6: minimum 15 animals SD 2.5; E7: minimum 3 animals SD 3.0; E8: minimum 7 animals SD 3.0; E9: minimum 15 animals SD 3.0; E10: minimum 3 animals SD 3.5; E11: minimum 7 animals SD 3.5; E12: minimum 15 animals SD 3.5. Models: (**A**) MD1: crop, birth farm, and W1OMG within CG, with birth season fitted outside CG; (**B**) MD2: birth season, birth farm, and W1OMG within CG, with crop fitted outside CG; (**C**) MD3: crop, birth farm, and yearling MG within CG, with birth season fitted outside CG; (**D**) MD4: crop, birth season, birth farm, and W1OMG within CG; (**E**) MD5: crop, birth farm, and yearling MG within CG; (**F**) MD6: crop, birth season, birth farm, and W1OMG within CG, with LAC fitted outside CG; (**G**) MD7: crop, birth season, birth farm, and weaning MG within CG; and (**H**) MD8: crop, birth season, birth farm, yearling MG, and weaning MG within CG. W1OMG: management group from birth to weaning of the first product; Weaning MG: animal management group from birth to weaning; Yearling MG: animal yearling management group; LAC: linear effect of cow age at calving; QAC: quadratic effect of cow age at calving; LAY: linear effect of yearling age of males on SC18 assessment; QAY: quadratic effect of yearling age of males on SC18 assessment.

For AFC, the models that included the yearling management group with MCGS = 3, 7, and 15 in SD 2 (MD3, MD5, and MD8) presented the highest residual variance values and lowest additive genetic variance, resulting in lower heritability estimates (around 0.06). The MD7 model (MCGS = 15) yielded the highest heritability estimate (0.39) (Figure 2).

The MD3 model, which included the yearling management group in the formation of the CG (with birth season as a fixed effect outside the group), presented a dispersion of 0.95 (the highest among the models evaluated), indicating lower inflation in the predictions of EBVs. In contrast, the MD4 model (MCGS = 15), which incorporated birth season and management group from birth to weaning of the first offspring, presented the lowest dispersion (0.64). There was no significant difference in dispersion between the different MCGS within the models. The MD8 model (MCGS = 3), which included both the yearling management group and the weaning management group, presented the highest accuracy value (0.90), while the MD2 (MCGS = 15), with birth season and management group from birth to weaning management group, yielded the lowest EBV accuracy (0.45). The lowest absolute bias (0.02) was observed for the MD4 model (MCGS = 7), and the highest bias (1.80) was found for the MD6 model (MCGS = 15).

Other measures of central tendency were also evaluated for AFC, such as mode and median, which showed values close to the mean for genetic variance, residual variance, and heritability, indicating a stable distribution of estimates across scenarios. Across the evaluated models (MD1–MD8), heritability estimates for AFC ranged from approximately 0.05 to 0.39, accuracy from 0.45 to 0.92, dispersion from 0.63 to 0.98, and bias from −0.37 to 1.80 (Figure 2). In general, models such as MD3, MD5, and MD8 showed high accuracy (0.86–0.92) and low heritability (0.05–0.07), whereas MD7 presented higher heritability (0.34–0.39) accompanied by increased bias (0.75–1.53). Notably, MD6 exhibited the highest bias values (>1.1).

**Figure 3:**
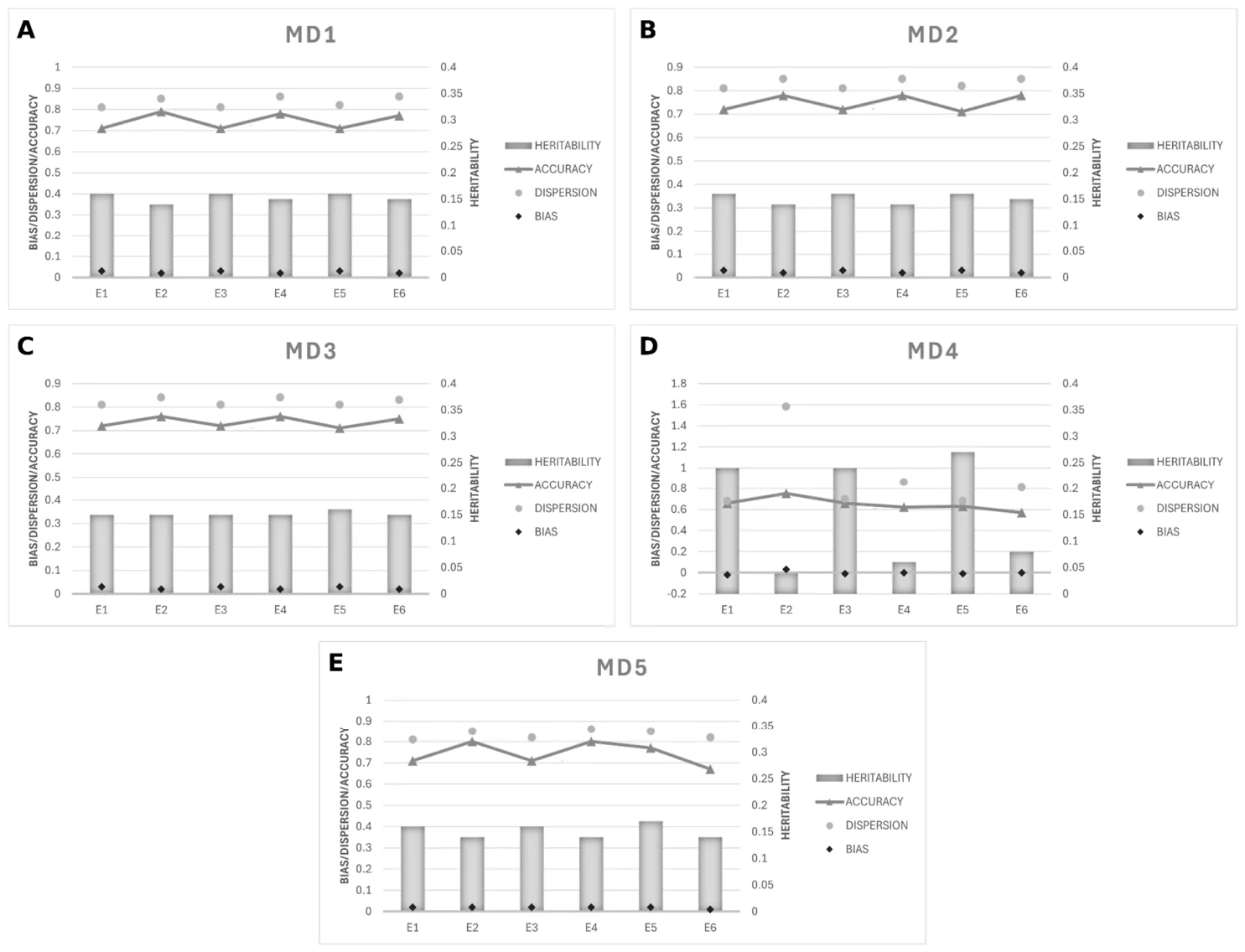
Estimates of heritability, accuracy, bias, and dispersion estimates across scenarios (E1–E6) for the MD1–5 models fitted for early pregnancy in Nellore cows. Scenarios: E1—minimum number of 3 animals per contemporary group (CG) and with phenotypic variability; E2—minimum 3 animals without variability; E3—minimum 7 animals with variability; E4—minimum 7 animals without variability; E5—minimum 15 animals with variability; and E6—minimum 15 animals without variability. Models: (**A**) MD1: crop, birth farm, and W1OMG within CG, with birth season fitted outside CG; (**B**) MD2: birth season, birth farm, and W1OMG within CG, with crop fitted outside CG; (**C**) MD3: crop, birth farm, and yearling MG within CG, with birth season fitted outside CG; (**D**) MD4: crop, birth season, birth farm, and W1OMG within CG; (**E**) MD5: crop, birth farm, and yearling MG within CG.

For EP, MD4, which included the weaning management group of the first calf within CG, presented the lowest values of residual variance (0.88) and genetic variance for MCGS = 15. The highest heritability estimate was observed with MD5 and MCGS = 15 (0.18), while the lowest value occurred for MCGS = 3 (0.14) (Figure 3). The exclusion of CGs due to low variability did not affect heritability estimates; however, the scenarios that excluded CGs due to low variability had slightly higher heritability estimates (on average 0.18) compared to scenarios that did not remove CGs due to variability (on average 0.13).

MD4 for EP presented the highest dispersion values (0.77, 0.78, and 0.74 in MCGS of 3, 7, and 15, respectively). The dispersion values averaged 0.79 for scenarios excluding CGs due to phenotypic variability and 0.89 for scenarios that did not exclude CG based on this criterion. The same model showed the lowest accuracy and bias values compared to the other models. In addition, there was no significant difference in EBV accuracy (mean 0.71 vs. 0.74) and bias (0.02) for scenarios that removed CG due to variability vs. those without exclusion due to variability.

The presence or absence of variability within the CG did not significantly alter the estimates of genetic parameters for EP. Considering the models evaluated (MD1–MD5), heritability ranged from 0.04 to 0.27, EBV accuracy from 0.57 to 0.80, dispersion from 0.68 to 1.58, and bias remained close to zero (between −0.02 and 0.03) in most scenarios. In general, models MD1, MD2, MD3, and MD5 presented moderate heritability estimates (0.14–0.17) associated with high EBV accuracy (0.71–0.80), while MD4 showed greater sensitivity to combinations of CG variability and minimum size, resulting in greater variation in dispersion (0.68–1.58) and heritability estimates (0.04–0.27).

**Figure 4:**
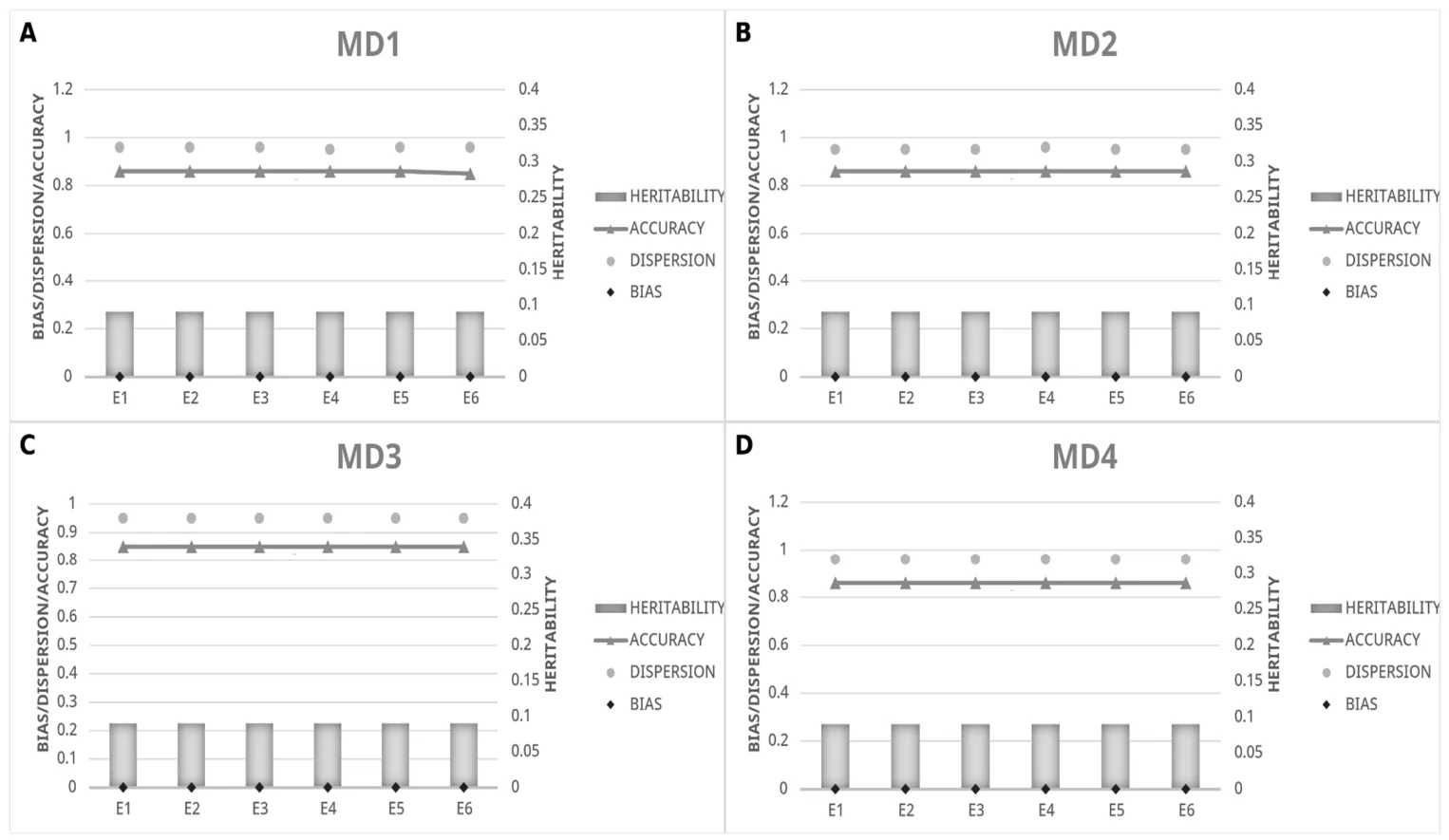
Heritability, accuracy, bias, and dispersion estimates across scenarios (E1–E6) for MD1–4 models estimated for the total number of calving at 53 months of age in Nellore cows. Scenarios: E1—minimum of 3 animals per contemporary group and with phenotypic variability; E2—minimum 3 animals without variability; E3—minimum 7 animals with variability; E4—minimum 7 animals without variability; E5—minimum 15 animals with variability; and E6—minimum 15 animals without variability. Models: (**A**) MD1: crop, birth season, birth farm, and yearling MG within CG, with LAC and QAC fitted outside CG; (**B**) MD2: birth season, birth farm, and yearling MG within CG, with crop fitted outside CG; (**C**) MD3: crop, birth farm, and yearling MG within CG, with birth season fitted outside CG; (**D**) MD4: crop, birth season, birth farm, and yearling MG within CG.

For NC53, the residual (mean of 0.72) and additive genetic (mean of 0.07) variances did not show significant differences between the different MCGS evaluated, as well as by the exclusion of CG due to variability in the models analyzed. Consequently, heritability estimates remained similar between the models evaluated (mean of 0.09) (Figure 4).

There were no significant differences in dispersion between the MCGS for NC53, nor in the presence or absence of variability within CG, which did not influence the EBV accuracy values (average 0.85) between the scenarios evaluated. The bias values were similar among the MCGS evaluated, with average values of −0.002 between models, under different MCGS and considering the presence or absence of phenotypic variability in the CG.

For trait NC53, the presence or absence of variability within CG and the difference in MCGS (MCGS = 3, 7, and 15 animals) did not significantly affect the estimates of genetic parameters, as illustrated in Figure 4. In all models evaluated (MD1–MD4), the bias was close to zero, the dispersion remained high and stable (0.95–0.96), accuracy was high (0.85–0.86), and heritability remained constant at 0.09.

**Figure 5:**
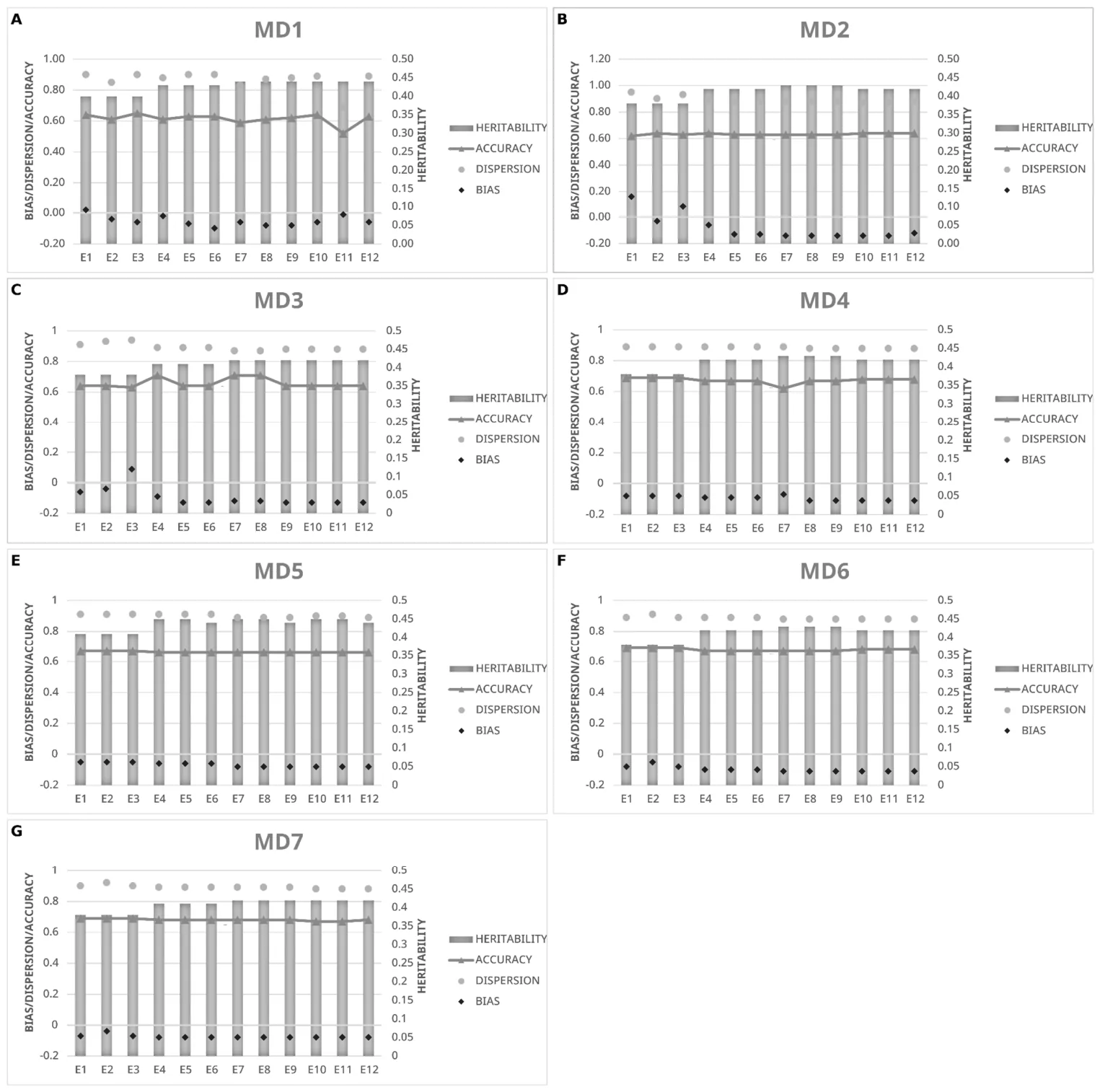
Estimates of heritability, accuracy, bias, and dispersion across scenarios (E1–E12) for the MD1–7 models estimated for scrotal circumference at 18 months in Nellore cattle. Scenarios: E1: minimum number of 3 animals per contemporary group (CG) and SD threshold of 2.0 for removing phenotypic outliers; E2: minimum 7 animals SD 2.0; E3: minimum 15 animals SD 2.0; E4: minimum 3 animals SD 2.5; E5: minimum 7 animals SD 2.5; E6: minimum 15 animals SD 2.5; E7: minimum 3 animals SD 3.0; E8: minimum 7 animals SD 3.0; E9: minimum 15 animals SD 3.0; E10: minimum 3 animals SD 3.5; E11: minimum 7 animals SD 3.5; E12: minimum 15 animals SD 3.5. Models: (**A**) MD1: crop, birth season, birth farm, and Yearling MG within CG, with LAY and QAY fitted outside CG; (**B**) MD2: birth season, birth farm, and Yearling MG within CG, with crop, LAY, and QAY fitted outside CG; (**C**) MD3: crop, birth farm, and Yearling MG within CG, with birth season, LAY, and QAY fitted outside CG; (**D**) MD4: crop, birth season, birth farm, and Yearling MG within CG; (**E**) MD5: crop, birth season, birth farm, Yearling MG, and Weaning MG within CG; (**F**) MD6: birth season, birth farm, and Yearling MG within CG, with crop fitted outside CG; (**G**) MD7: crop, birth farm, and Yearling MG within CG, with birth season fitted outside CG.

For SC18, MD1, which included birth cohorts, season, and farm of birth, and management group at weaning when forming the CGs and fixed effects outside the CG, including the linear and quadratic age at weaning, presented the lowest values of residual variance (3.67) and additive genetic variance (2.75). The highest heritability estimate (0.42) was observed for the MD5 model (MCGS = 3 and 7), a model that differed by including the weaning management group in the formation of the CG. The lowest heritability estimate (0.40) was recorded for MD7 (MCGS = 3, 7, and 15), a model that considered the birth season as a fixed effect outside the CG (Figure 5).

The lowest dispersion was observed in the MD1 model with MCGS = 7 (0.83) for SC18. Regarding EBV accuracy, MD1 (MCGS = 7) showed the lowest value (0.59), whereas MD3 achieved the highest accuracy (0.71), slightly outperforming MD4, MD6, and MD7 (0.68). Bias remained low across all scenarios, ranging from -0.11 in MD1 (MCGS = 3) to -0.03 in MD2 (MCGS = 7).

The variation in SD between scenarios did not impact the estimates of genetic parameters. Heritability estimates ranged from 0.40 to 0.42 and accuracies from 0.59 to 0.71, with high dispersion (around 0.83 to 0.95) and low bias (−0.03 to ×0.11). In general, models with a more complex CG structure, which included a greater number of environmental effects (MD3, MD4, MD5, and MD7), presented slightly higher heritability estimates (greater than or equal to 0.41) without a significant reduction in EBV accuracy.

### 3.4. Consistency of EBV Rankings

The EBV rankings showed high consistency across the evaluated scenarios. For the top 1% of males and top 20% of females, the mean concordance across pairwise scenario comparisons was 94.8%, whereas the mean concordance in the ranking of all animals was 95.4%. Across traits, the concordance in the ranking of all animals ranged from 91.8% for AFC to 98.6% for NC53, indicating that changes in outlier-removal criteria and MCGS generally resulted in limited changes in animal ranking. Although some individual comparisons showed concordance below 90%, the overall consistency among scenarios remained high. The detailed concordance results for AFC, EP, NC53, and SC18 are presented in Supplementary Tables S5, S6, S7 and S8, respectively.

## 4. Discussion

The four traits evaluated in this study were selected based on their relevance to breeding objectives and their practical applicability in commercial beef cattle production. Age at first calving (AFC) and scrotal circumference at 18 months (SC18) are commonly included in genetic improvement programs, whereas heifer pregnancy (EP) and the number of calves born by 53 months of age (NC53) have gained attention as indicators of sexual precocity and longevity, respectively. In addition, these traits encompass different types of response variables, including continuous, binary, and categorical traits, allowing the performance of alternative CG definitions and quality-control criteria to be assessed across diverse evaluation scenarios.

The proper definition of CG is crucial for obtaining accurate and reliable EBVs. Thus, it is essential to consider environmental factors in genetic models, reducing the estimated residual variance [5]. The correct definition of CG as a fixed effect may reduce biases and improve heritability estimates in beef cattle populations under intense selection [7]. Quality control methods, choice of effects in CG formation, MCGS, variability, and appropriate SD are decisive in genetic estimates and animal classification.

Actually, the impact of these factors on genetic parameters is still poorly explored, since much of the literature assumes “standard” values for MCGS, standard deviation, and cleaning criteria, applying them to different populations and traits without a systematic assessment of their consequences on variances, heritabilities, and accuracies [19,20]. Literature is mainly focused on model choice (e.g., random effects and covariance structure), while data filtering procedures and fixed effect definition are described briefly, even though they can introduce important biases and alter connectedness between CG [20,21]. Explicitly investigating how these elements of preprocessing and fixed effects specification affect genetic estimates, as proposed in this study, is essential for developing more effective strategies for defining and using CG and, consequently, improving the reliability of genetic evaluations.

The removal of outliers based on phenotypic SD inside CG is generally used in genetic evaluation to ensure data consistency, but the SD threshold for outlier removal is usually determined empirically, without robust studies defining the ideal value to maximize the accuracy of EBVs [14]. Studies that evaluated strategies for detecting and treating outliers in production and genetic evaluation data showed that both excessive removal and retention of many extreme values can distort variance estimates and reduce the accuracy and unbiasedness of predicted EBVs, reinforcing that the impact of the cutoff criterion depends on the data structure, statistical model, and proportion of removed records [13,21,22,23].

Furthermore, although the ±2.0 and ±2.5 SD thresholds may initially appear overly restrictive due to the larger proportion of records excluded, these scenarios were included to evaluate the sensitivity of genetic parameter estimates to different levels of data editing. Assessing more extreme thresholds provides valuable insight into how increasingly stringent CG cleaning criteria affect heritability estimates, prediction accuracies, bias, and the overall robustness of genetic evaluations. In addition, the use of similar thresholds has been previously reported in beef cattle studies evaluating CG formation and data editing strategies. For example, Silva et al. [9] investigated outlier removal using thresholds of ±2.0, ±2.5, ±3.0, and ±3.5 standard deviations in Nellore cattle genetic evaluations, demonstrating that a range of editing criteria can substantially influence genetic parameter estimates and selection responses. For reproductive traits, extreme phenotypes may represent genuine biological variation rather than incorrect observations.

Although the MCGS and standard deviation thresholds were initially chosen based on previous studies [24], these scenarios also have biological and statistical implications that motivated their evaluation. From a statistical point of view, increasing the MCGS, to 15, for example, generally improves the stability and accuracy of CG effect estimates by reducing sampling variation and the influence of individual observations. Conversely, more restrictive CG definitions also lead to the exclusion of a larger number of animals, potentially reducing the representativeness of the analyzed population. Therefore, the thresholds of 3, 7, and 15 animals were used to evaluate the balance between data retention and statistical robustness.

From a biological perspective, reproductive performance is strongly influenced by management practices, which may also affect variances estimates [25]. For example, animals with lower reproductive efficiency may not have the opportunity to express puberty or reproductive success when exposed to fixed breeding seasons or management decisions that limit reproductive opportunities. Consequently, stringent exclusion criteria may disproportionately remove biologically informative records and alter the real phenotypic and genetic variability [26]. The scenarios investigated in this study were therefore intended to quantify how different levels of data editing affect both population structure and genetic parameter estimate.

### 4.1. Connectedness Between Contemporary Groups

Connectedness between CG is a fundamental element in ensuring comparability between animals and reliability in genetic estimates. High levels of connectedness reduce bias and increase accuracy, allowing EBVs to be evaluated consistently across different herds, management practices, and birth cohorts [10,11,16,27,28]. Most models showed high connectedness (>90%) for all traits, evidencing a well-interconnected genetic structure between the formed groups. The lowest proportions of connectedness were observed for continuous traits (AFC and SC18), although the highest number of genetic ties was observed for NC53 and SC18. Overall, there was little variation between models and MCGS, indicating that these factors had little influence on connectedness between groups. Of the total animals evaluated, 49.43% were from artificial insemination, which may have contributed to the high levels of connectedness observed, since proven bulls used in multiple herds promote genetic ties between different groups and properties. Furthermore, the large number of available phenotypes and the dense kinship structure of the data could contribute to this result.

In genetic evaluations with reduced connectedness, accuracy tends to be compromised, especially in populations with truncated or incomplete pedigrees. In addition, recent studies in beef cattle indicate that adequate levels of connectedness between CG are essential for leveraging genomic information effectively, as genomic predictions depend on establishing reliable relationships between genotyped and non-genotyped animals across management groups. Well-connected CG facilitates the integration of genomic data into single-step Genomic Best Linear Unbiased Prediction (ssGBLUP), improving the accuracy of predictions for animals with incomplete pedigree records [20,29].

Connectedness across CG is also important in the context of genomic evaluations and genotype-by-environment (G × E) interaction studies. Adequate connection between CG helps to use genomic information effectively, ensuring that genetic relationships are accurately estimated across different environments [30]. This is essential for detecting proper G×E interactions; a well-connected data structure enables more powerful G × E analyses and improves the accuracy of genomic predictions for traits sensitive to environmental variation [31].

Particularly noteworthy was the smaller reduction in the number of phenotypes and CG for NC53 and SC18 when compared to the other traits. The denser kinship structure for these traits makes them less sensitive to increases in minimum group size, preserving the volume of useful information for genetic evaluations even in more restrictive scenarios [11,20]. These results corroborate that robust genetic connection networks minimize information loss when forming CG, allowing fair comparisons between individuals even when the data structure is subject to more rigorous cleaning criteria [20,26].

In populations with smaller herds or limited use of common sires across herds, connectability may decrease, making it more difficult to separate genetic and environmental effects. Reduced connectedness can lead to lower accuracy of breeding value predictions [32,33], increased prediction error variance, and less reliable comparisons of animals across herds or management groups. In extreme cases, breeding values may become inflated or deflated due to incomplete separation of CG and genetic effects. Therefore, the optimal editing criteria are likely population-specific and should balance data quality, data retention, and genetic connectedness.

### 4.2. Estimates of Variances and Heritability, Dispersion, Accuracy and Bias

Heritability in the strict sense indicates the extent to which phenotypes are influenced by genes transmitted by parents, thus determining the degree of similarity between relatives. The predictive quality of models for genetic prediction can be accessed using metrics proposed by Legarra and Reverter [17], which include dispersion, bias, and accuracy of EBV. Accuracy reflects the expected correlation between the true breeding value (TBV) and the estimated breeding value (EBV). As the TBV cannot be directly observed in practice, accuracy is indirectly inferred and represents the reliability of EBV for ranking individuals and predicting genetic merit within a population, as defined by Legarra and Reverter [17]. Thus, accuracy serves as a key criterion for comparing genetic evaluation models and maximizing genetic progress. It ranges from 0 to 1, and as it approaches 1, the greater the confidence in the estimates. Dispersion indicates the degree of inflation, and it is expected to be 1 if there is not over (inflation) or under (deflation) dispersion. Dispersion may assume values below 1 (overdispersion), which means that the estimates are more dispersed than expected, i.e., the estimated values present more variation around the actual value than they should or values above 1 (deflation), where the estimates are less dispersed than expected, i.e., the estimated values are very close to each other of the estimates, this generally occurs with small or old data [17]. Deviations of up to 5% from a unit are considered good estimates, while deviations of up to 15% are considered acceptable [17,34]. In this study, bias was indirectly assessed as the difference between the mean EBVs of the animals in the validation population predicted using the full and reduced datasets [17].

### 4.3. Age at First Calving

Regarding the heritability for AFC, our estimates (across models and scenarios) are within the range reported in the literature for this trait in Nellore cattle. Costa et al. [5] found heritability of 0.08 using CG formed by herd, year, and season of birth, while Boligon and Albuquerque [35] reported values between 0.16 and 0.20. Recently, Brunes et al. [36] estimated heritability of 0.11 for age at first conception in Nellore cattle, observing favorable genetic correlations with growth and carcass traits, which reinforces the viability of selection for sexual precocity without compromising weight development.

The inclusion of the yearling management group in the present study resulted in an increase in residual variance and, consequently, lower heritability estimates for AFC (MD2, MD3, MD5, and MD8), whereas models that excluded this effect from the CG definition yielded higher heritabilities (MD7). This reduction in heritability does not necessarily indicate a loss of accuracy. These lower values may reflect a more appropriate partitioning of phenotypic variance, as part of the environmental variation that could be confounded with additive genetic variance. This interpretation may be further supported by the slightly higher accuracies observed in models including the yearling management group, without substantial changes in bias. Considering that the critical period for reproductive development in females occurs between four and nine months of age [37], the yearling management group represents a key environmental factor influencing AFC and, therefore, plays an important role in the estimation of genetic parameters for this trait in Nellore cattle [36,38].

The highest bias observed for MD6 with MCGS = 15 (1.80) indicates a substantial overestimation of breeding values, suggesting that this model structure may not adequately account for the sources of variation affecting AFC. Such inflation may reduce the reliability of selection decisions by favoring animals whose genetic merit is overpredicted [16,38]. The MD6 model was the only model that included both effects, the management group of the first product (inside CG) and the linear effect of age at dam as a covariate, the combination of both effects in the same model seems to produce higher bias.

The superiority of models presenting lower bias and dispersion values closer to unity indicates a better fit to the genetic architecture of the evaluated traits. This suggests that appropriate model specification contributes to more robust and consistent breeding value predictions, improving the reliability of genetic evaluations.

### 4.4. Early Pregnancy

Recent studies in Nellore cattle highlight the importance of early pregnancy as a selection criterion for reproductive efficiency, given its favorable genetic correlations with stayability and accumulated productivity traits [39,40]. Some herds expose their females earlier to reproduction, at around 14 months of age, given the opportunity for the cows to express this trait.

The inclusion of the management group at weaning of the first progeny (MD4) had little influence on the genetic parameters for EP, in contrast to what was observed for AFC. Although some sensitivity of MD4 to different MCGS was observed, with heritability estimates ranging from 0.14 (MCGS = 3) to 0.18 (MCGS = 15), these differences were small, indicating that the MCGS had limited impact on the accuracy and stability of genetic parameter estimates for EP (Figure 3). Additionally, heritability estimates did not vary significantly when quality control excluded CGs based on variability. Kluska et al. [40] reported a heritability of 0.37 for EP, higher than those observed in the present study, which may be attributed to differences in model structure and CG definition, as their CGs were formed by concatenating farm, year, and season of birth effects in Nellore cattle.

The accuracies observed for EP (0.60 to 0.75) are consistent with the literature, which typically ranges from moderate to high depending on the amount of phenotypic information available [41]. The low bias found in all models (−0.002 to 0.03) indicates that the systematic effects were well estimated and that EBVs are not systematically biased. Regarding dispersion, it followed a pattern of overdispersion (b < 1), which is often reported in genetic evaluations, particularly for traits with lower heritability [17].

### 4.5. Number of Calving at 53 Months of Age

For NC53, heritability was similar across models, at 0.09. These estimates are lower than those reported by Neves et al. [42], who found heritability of 0.16 for NC53 in Nellore cattle, and by Guarini et al. [43], who estimated heritability of 0.22 with additive variance of 0.29 and residual variance of 0.10. Recent studies also report low heritability estimates for other longevity traits, as stayability and number of calving in beef cattle. Silva et al. [44] found heritabilities between 0.05 and 0.08 for stayability in cross breeds cattle, in addition, Passafaro et al. [12] reported heritability of 0.10 for productive longevity in a multibreed beef cattle population.

These differences may be related to the composition of the CG, other factors included in the model and particularities of the evaluated populations. In the present study, the stability of the estimates suggests that the evaluated models and the Bayesian approach were effective in capturing the genetic variability for NC53 in a consistent and robust manner. The low heritability observed for NC53 is consistent with previous studies on longevity-related traits in Nellore cattle, which are typically characterized by low heritability due to their complex biological nature and strong dependence on management and environmental conditions [12,43,44]. This reinforces the importance of careful adjustments to the statistical models to adequately capture the environmental effects that may affect the traits in order to improve the accuracy of EBVs.

Accuracy was high and stable across models, while bias remained close to zero, indicating no bias in the predictions. Overall, the dispersion estimates did not indicate the presence of inflated results, remaining close to 1.0 across models and MCGS.

### 4.6. Scrotal Circumference at 18 Months of Age

Heritability for SC18 presented a low range between models (0.41–0.44). These findings are consistent with previous studies, such as Laureano et al. [45], who estimated heritability of 0.42 in Nellore cattle, while Boligon et al. [46] reported values between 0.35 and 0.37 for Nellore at 18 months. Nascimento et al. [47], found heritabilities ranging from 0.60 to 0.85 for scrotal circumference at 18 months in Brahman cattle, using genomic information.

Accuracy remained generally stable across models; however, MD1 (scenario E11) and MD3 (scenarios E4–E8) presented some oscillation. When comparing MD1 and MD4, which differ only by the inclusion of yearling age (linear and quadratic) as a covariate in MD1. MD1 consistently showed slightly lower accuracy across MCGS (0.59–0.63) than MD4 (0.66–0.68), despite similar heritability estimates (MD1 = 0.43; MD4 = 0.41). These results suggest that the inclusion of age as a covariate did not significantly impact the predictive performance for SC18 in this population. For SC genetic evaluations, it is common to use the age at which the SC measurement was performed as a covariate in the model or the animal’s weight at the assessment [48]. Our results showed no evidence of differences between models that included or did not include this covariate (MD1–MD3 vs. MD4–MD7). In general, it is recommended to test this covariate (linear and quadratic effects) before performing genetic evaluations, as we did in this study, and even if it is not significant, it is interesting to keep at least the linear effect in the model due to the known importance of this covariate for SC. The bias, in general, remained close to zero, producing reliable predictions across models. Overall, dispersion estimates were stable across models and scenarios and did not indicate the presence of inflated results, with bias values close to zero. Therefore, dispersion did not meaningfully vary among the evaluated models and scenarios. In contrast, prediction accuracy showed small but relevant differences.

It is important to highlight that higher heritabilities did not necessarily imply a better model, especially for these reproductive traits, for which heritability is often low due to both substantial environmental influence and the long-term selection for reproductive efficiency in commercial populations, which may have reduced the additive genetic variance available for these traits [24]. The main decision criteria about models or scenarios should be based on a set of other factors such as connectability, accuracy, bias, and dispersion.

### 4.7. EBVS Ranking

Although previous studies in Nellore cattle have recommended CG with at least 15 animals to maximize genetic gains in growth traits [20], and studies with pigs warn that excessive fragmentation can affect ranking stability [9], the results of the present study for reproductive traits showed high consistency in animal classification even with reduced MCGS (MCGS = 3). This suggests that, for the reproductive and longevity traits in the beef cattle population evaluated here, smaller CG may be efficient, reducing the need for a high number of animals per CG without compromising selection stability [41].

The choice of the MCGS should therefore balance the accuracy of genetic estimates, the consistency of EBV rankings, and practical operational feasibility, considering the specific population structure and selection objectives of the breeding program.

### 4.8. Study Limitations and Future Perspectives

This study has some limitations that should be considered when interpreting the results. First, genomic information was not included in the genetic evaluations. Although genomic information could potentially provide additional information on genetic relationships among animals, the main objective of this study was to evaluate the effects of different contemporary group structures on variance component estimation using traditional pedigree information. Therefore, the absence of genomic information does not preclude interpretation of the main findings within the specific scope and objectives of this study. Previous studies have also reported that the inclusion of genomic information did not substantially change variance component estimates in some cattle populations [13,14].

The study population was predominantly derived from pasture-based beef cattle systems, which may involve greater environmental heterogeneity than more controlled intensive production systems [23]. Consequently, the structure and definition of contemporary groups may behave differently under confinement or other production conditions. Future studies should therefore evaluate contemporary group definitions across different production systems and larger populations. In addition, studies involving multi-breed populations could help assess the consistency and broader applicability of these findings across beef cattle breeding programs.

Future research could also explore data-driven approaches to contemporary group formation. High-throughput phenotyping, automated monitoring, and machine learning methods may provide opportunities to identify environmental heterogeneity and refine contemporary group definitions as new data become available. Such approaches could complement traditional criteria based on management and production information and potentially improve the adaptability of genetic evaluations across different production environments.

## 5. Conclusions

The results of this study reinforce the importance of carefully structured contemporary groups in the genetic evaluation of reproductive traits. The proper definition of CG, combined with the analysis of connectedness, bias, dispersion, and accuracy, is essential to ensure consistent and reliable genetic estimates. The high connectedness between groups and models evaluated reflected the good structure of the data. The fixed effects evaluated, season of birth, birth cohorts, farm, and management group, affected heritability and EBV estimates for some traits, demonstrating the critical importance of including environmental factors in genetic models. Although the different standard deviations for removing outliers in continuous traits and the presence of variability in categorical traits did not cause significant changes in the results, the inclusion of all environmental effects in the models substantially improved genetic evaluation quality. For age at first calving, models that incorporated management groups at later life stages, particularly the yearling management group, produced more accurate and less biased EBVs compared with models that did not account for this source of variation.

In addition, the minimum size of animals per contemporary group did not have a major influence on the results. However, a minimum contemporary group size of three animals consistently demonstrated values closer to the ideal benchmarks for bias (closer to zero), dispersion (closer to 1), and accuracy (closer to 1) across all evaluated traits and statistical models, regardless of data quality scenario. Furthermore, this group size showed higher correlations between scenarios, indicating greater consistency and stability in genetic evaluation outcomes. These findings demonstrate that to our population and data, smaller groups, when well-structured with appropriate environmental effects, can maintain the quality of genetic evaluations, optimizing selection without compromising the reliability of estimates. Reproductive traits with different heritabilities and genetic architectures, as well other population structures, may respond differently to outlier removal criteria and minimum group size thresholds.

Defining optimal contemporary groups for each trait is an attractive concept, but its implementation requires careful evaluation of trade-offs between statistical rigor and practical feasibility, particularly regarding computational costs and data availability in commercial breeding programs. Furthermore, the parameters evaluated in this study are highly dependent on population-specific traits, such as population size, genetic variability and allele frequencies. Therefore, our findings may be cautiously extrapolated to other Nellore or Zebu populations with similar genetic backgrounds. Future studies with larger cohorts across different breeds and production systems, combined with functional validation of genetic mechanisms, are needed to extend these results and achieve better understanding of optimal contemporary group formation. This will enable the development of data quality standards and selection approaches to improve reproductive efficiency and genetic gain in beef cattle breeding programs.

## Data Availability

The datasets presented in this article are not readily available because they are from Gensys and can only be accessed with the company’s authorization. Requests to access the datasets should be directed to the corresponding author (Thaise Pinto de Melo).

## Supplementary Materials

The following supporting information can be downloaded at: https://www.mdpi.com/article/10.3390/ani16203222/s1, Table S1. Descriptive statistics of the evaluated models (MD1–8) according to the minimum number of animals per contemporary group for age at first calving in Nellore heifers. Table S2. Descriptive statistics of the evaluated models (MD1–5) according to the minimum number of animals per contemporary group of early pregnancy in Nellore heifers. Table S3. Descriptive statistics of the evaluated models (MD1–4) according to the minimum number of animals per contemporary group for the number of calvings at 53 months of age in Nellore females. Table S4. Descriptive statistics of the evaluated models (MOD1–7) according to the minimum number of animals per contemporary group for scrotal circumference at 18 months in Nellore males. Table S5. Concordance in EBV ranking across the evaluated scenarios for age at first calving (AFC) in Nellore heifers. The upper diagonal presents the percentage of common animals among the top 1% of males and top 20% of females, whereas the lower diagonal presents the percentage concordance in the ranking of all animals. Table S6. Concordance in EBV ranking across the evaluated scenarios for early pregnancy (EP) in Nellore heifers. The upper diagonal presents the percentage of common animals among the top 1% of males and top 20% of females, whereas the lower diagonal presents the percentage concordance in the ranking of all animals. Table S7. Concordance in EBV ranking across the evaluated scenarios for number of calvings at 53 months of age (NC53) in Nellore females. The upper diagonal presents the percentage of common animals among the top 1% of males and top 20% of females, whereas the lower diagonal presents the percentage concordance in the ranking of all animals. Table S8. Concordance in EBV ranking across the evaluated scenarios for scrotal circumference at 18 months (SC18) in Nellore males. The upper diagonal presents the percentage of common animals among the top 1% of males and top 20% of females, whereas the lower diagonal presents the percentage concordance in the ranking of all animals. Table S9. Number of genetic links, mean percentage of animals (AP), and connected contemporary groups (CCc) for the evaluated models (MD1–8) for age at first calving (AFC). Table S10. Number of genetic links, mean percentage of animals (AP), and connected contemporary groups (CG) for the evaluated models (MD1–5) for early pregnancy (EP). Table S11. Number of genetic links, mean percentage of animals (AP), and connected contemporary groups (CGc) for the evaluated models (MD1–4) for the number of calvings by 53 months of age. Table S12. Number of genetic links, mean percentage of animals (AP), and connected contemporary groups (CGc) for the evaluated models (MD1–7) for scrotal circumference (SC18).
