# Supplementary material for: Comparison of Models Fitting Alternative Contemporary Group Definition and Data Editing Procedures in the Genetic Evaluations of Reproductive Performance and Cow Longevity Traits in Nellore Cattle
Source: Animals (Basel). 2026 Oct 13;16(20):3222. doi: 10.3390/ani16203222 (PMC13700584; doi:10.3390/ani16203222)
Supplement: Supplementary file 1 [file animals-16-03222-s001.zip › animals-4518049-supplementary.pdf]

Article

Comparison of models fitting alternative contemporary group definition and data editing procedures in the genetic evaluations of reproductive performance and cow longevity traits in Nellore cattle

Andreia Kaspary Zwirtes[1], Daniele Alves de Oliveira[1], Saymon Silva Rocha[1], Delvan Alves Silva[2], Luiz Fernando Brito[3], Larissa Bordin Temp[4], Nathalia Pereira Seixas[1], Roberto Carvalheiro[5], Lucia Galvão de Albuquerque[4], and Thaise Pinto de Melo [1,*]

[1] Animal Science Department, Federal University of Santa Maria, Santa Maria, RS, Brazil; andreiakasparyzoot@gmail.com (AKZ); daniele.alves@acad.ufsm.br (DAO); saymon.rocha@acad.ufsm.br (SSR); nathaliaaseixasp@gmail.com (NPS)

[2] Department of Animal Science, Federal University of Viçosa (UFV), Viçosa, MG, Brazil; delvan.silva@ufv.br (DAS)

[3] Department of Animal Sciences, Purdue University, West Lafayette, United States; britol@purdue.edu (LB)

[4] Department of Animal Science, São Paulo State University (UNESP), Jaboticabal, SP, Brazil; larissabordintemp@gmail.com (LT); galvao.albuquerque@unesp.br (GA)

[5] Commonwealth Scientific and Industrial Research Organisation (CSIRO) – Hobart, Tasmania, Australia; roberto.carvalheiro@unesp.br (RC)

* Correspondence: thaise.melo@ufsm.br

Supplementary Materials

Table S1. Descriptive statistics of the evaluated models (MD_{1-8}) according to the minimum number of animals per contemporary group for age at first calving in Nellore heifers.

MD	MCGS	N	%REDan	CG	%REDCG	$\bar{X}$ AFC (SD)
	3	199512	2.52	5850	20.11	1014 (129.94)
MD1	7	194479	6.18	4674	25.52	1014 (129.99)
	15	182451	8.55	3481	40.49	1012 (130.21)
	3	196191	4.28	8035	24.30	1013 (130.48)
MD2	7	187801	10.98	6082	33.97	1011 (130.70)
	15	167187	14.78	4016	50.02	1007 (131.16)
	3	201605	0.41	2922	6.25	1016 (129.48)
MD3	7	200781	1.72	2739	12.28	1016 (129.50)

	15	197319	2.13	2403	17.76	1016 (129.61)
MD4	3	195608	5.10	8555	27.17	1014 (130.41)
	7	185636	12.18	6231	36.15	1012 (130.28)
	15	163032	16.65	3979	53.50	1008 (130.04)
MD5	3	200028	0.98	4380	10.10	1016 (129.57)
	7	198073	3.96	3937	19.49	1015 (129.54)
	15	190236	4.90	3170	27.63	1015 (129.63)
MD6	3	195608	5.10	8555	27.17	1014 (130.41)
	7	185636	12.18	6231	36.15	1012 (130.28)
	15	163032	16.65	3979	53.50	1008 (130.04)
MD7	3	187181	9.24	12190	36.94	1012 (130.83)
	7	169885	19.48	7687	46.16	1009 (131.20)
	15	136790	26.92	4139	66.05	1006 (132.62)
MD8	3	194163	4.22	10843	15.68	1015 (130.59)
	7	185963	23.02	9143	46.21	1015 (130.14)
	15	143161	26.27	4918	54.64	1018 (128.32)

MCGS = minimum number of animals per contemporary group; N = average number of animals for each minimum size; $\%RED_{ani}$ = average percentage reduction in the number of animals across groups with different minimum sizes (3–7, 7–15, and 3–15) relative to the total; CG = average number of contemporary groups per minimum size and model; $\%RED_{GC}$ = average percentage reduction in the number of contemporary groups relative to the total; $\bar{X}_{AFP}$ = phenotypic mean of age at first calving; SD = phenotypic standard deviation.

Table S2. Descriptive statistics of the evaluated models ($MD_{1–5}$) according to the minimum number of animals per contemporary group of early pregnancy in Nellore heifers.

MD	MCGS	N	%REDan	CG	%REDCG	N (0)	%RED(0)	N (1)	%RED(1)
MD1	3	302217	0.1	3417	2.9	263942	0.13	38331	0.25
	7	301780	1.1	3319	9.4	263601.5	1.09	38235	1.56
	15	298321	1.3	3007	12.0	260731.5	1.22	37639.5	1.80
MD2	3	302391	0.1	3403	2.9	264114.5	0.13	38333	0.25
	7	301955	1.1	3305	9.3	263774	1.08	38237	1.48
	15	298559	1.3	2999	11.9	260938	1.20	37671	1.73
MD3	3	312887	0.01	2289	0.3	274054	0.00	38890	0.06
	7	312853	0.44	2283	5.2	274043.5	0.41	38866.5	0.65
	15	311481	0.45	2165	5.4	272918	0.41	38613.5	0.71
MD4	3	110979	4.7	4577	25.9	87766	5.07	23212.5	3.16
	7	105798	11.5	3391	35.2	83319	12.17	22478.5	9.14
	15	93601	15.7	2199	52.0	73177.5	16.62	20423.5	12.02
MD5	3	277837	0.20	8403	1.20	240514	0.10	37380.5	0.83
	7	277279	8.15	8302	23.39	240264	7.44	37072	12.77
	15	254679	8.34	6360	24.31	222393.5	7.53	32336.5	13.49

MCGS = minimum number of animals per contemporary group; N = average number of animals for each minimum size; $\%RED_{an}$ = average percentage reduction in the number of animals across groups with different minimum sizes (3–7, 7–15, and 3–15) relative to the total; CG = average number of contemporary groups per minimum size and model; $\%RED_{CG}$ = average percentage reduction in the number of contemporary groups relative to the total; Exclusion = with and without variability.

Table S3. Descriptive statistics of the evaluated models ($MD_{1\text{–}4}$) according to the minimum number of animals per contemporary group for the number of calvings at 53 months of age in Nellore females.

MD	MCGS	N	%REDan	CG	%REDCG	N (0)	%RED(0)	N (1)	%RED(1)	N (2)	%RED(2)	N (3)	%RED(3)
	3	352975	0.1	4137	2.7	164404	0.1	81276	0.2	103429	0.1	3929	0.1
MD1	7	352453	1.3	4025	10.0	164162	1.5	81137	1.2	103293	1.0	3924	1.0
	15	347973	1.4	3624	12.4	161715	1.6	80158	1.4	102267	1.1	3883	1.2
	3	353049	0.1	4120	2.7	164447	0.1	81300	0.2	103436	0.1	3929	0.1
MD2	7	352527	1.3	4008	9.9	164205	1.5	81161	1.2	103300	1.0	3924	1.0
	15	348084	1.4	3610	12.4	161781	1.6	80189	1.4	102281	1.1	3883	1.2
	3	354626	0.0	2695	0.0	165563	0.0	81545	0.0	103648	0.0	3934	0.0
MD3	7	354626	0.5	2695	5.6	165563	0.6	81545	0.5	103648	0.4	3934	0.2
	15	352842	0.5	2543	5.6	164566	0.6	81126	0.5	103274	0.4	3927	0.2
	3	352975	0.1	4137	2.7	164404	0.1	81276	0.2	103429	0.1	3929	0.1
MD4	7	352453	1.3	4025	10.0	164162	1.5	81137	1.2	103293	1.0	3924	1.0
	15	347973	1.4	3624	12.4	161715	1.6	80158	1.4	102267	1.1	3883	1.2

MCGS = minimum number of animals per contemporary group; N = average number of animals for each minimum size; %REDan = average percentage reduction in the number of animals across groups with different minimum sizes (3–7, 7–15, and 3–15) relative to the total; GC average number of contemporary groups per minimum size and model; %REDCG = average percentage reduction in the number of contemporary groups relative to the total; Exclusion = with and without variability.

Table S4. Descriptive statistics of the evaluated models (MD_{1-7}) according to the minimum number of animals per contemporary group for scrotal circumference at 18 months in Nellore males.

MD	MCGS	N	%REDan	CG	%REDCG	$\bar{X}$ SC18 (SD)
MD1	3	512028	0.16	6499	2.87	26.67 (3.77)
	7	511218	1.36	6313	10.28	26.67 (3.77)
	15	504262	1.52	5664	12.86	26.67 (3.77)
MD2	3	512150	0.16	6480	2.82	26.67 (3.77)
	7	511341	1.34	6297	9.99	26.67 (3.77)
	15	504492	1.50	5664	12.52	26.67 (3.77)
MD3	3	518155	0.00	4330	0.00	26.66 (3.77)
	7	518155	0.55	4330	5.82	26.66 (3.77)
	15	515282	0.55	4078	5.82	26.66 (3.77)
MD4	3	512028	0.16	6499	2.87	26.67 (3.77)
	7	511218	1.36	6313	10.28	26.67 (3.77)
	15	504262	1.52	5664	12.86	26.67 (3.77)
MD5	3	493168	0.00	15017	0.00	26.76 (3.77)
	7	493168	7.52	15017	20.34	26.76 (3.77)
	15	456082	7.52	11963	20.34	26.77 (3.77)
MD6	3	512150	0.16	6480	2.82	26.67 (3.77)
	7	511341	1.34	6297	10.05	26.67 (3.77)
	15	504492	1.50	5664	12.59	26.67 (3.77)
MD7	3	518154	0.00	4330	0.00	26.65 (3.77)
	7	518154	0.55	4330	5.82	26.65 (3.77)
	15	515282	0.55	4078	5.82	26.66 (3.77)

MCGS = minimum number of animals per contemporary group; N: average number of animals for each minimum size; %REDan = average percentage reduction in the number of animals across groups with different minimum sizes (3–7, 7–15, and 3–15) relative to the total; CG = average number of contemporary groups per minimum size and model; %REDCG = average percentage reduction in the number of contemporary groups relative to the total; $\bar{X}$ SC18 = phenotypic mean of scrotal circumference; SD = phenotypic standard deviation.

Table S5. Concordance in EBV ranking across the evaluated scenarios for age at first calving (AFC) in Nellore heifers. The upper diagonal presents the percentage of common animals among the top 1% of males and top 20% of females, whereas the lower diagonal presents the percentage concordance in the ranking of all animals.

Datasets[(1)]	Datasets[(1)]											
	E1	E2	E3	E4	E5	E6	E7	E8	E9	E10	E11	E12
E1 (2.0-3)	100.00	96.66	91.28	93.38	92.68	88.28	91.96	91.34	87.32	91.25	89.98	87.35
E2 (2.0-7)	95.33	100.00	92.01	92.61	93.52	89.49	91.37	92.19	88.53	90.76	90.84	87.93
E3 (2.0-15)	90.44	90.77	100.00	88.05	89.24	93.69	87.09	87.52	92.35	86.28	86.74	91.81
E4 (2.5-3)	93.29	92.61	87.46	100.00	96.69	90.31	96.20	95.41	89.36	95.80	93.63	88.97
E5 (2.5-7)	92.86	92.88	88.24	95.67	100.00	91.31	95.47	96.09	90.47	94.05	94.95	90.11
E6 (2.5-15)	87.29	89.05	92.16	91.00	90.92	100.00	89.06	89.95	96.42	88.57	88.97	95.28
E7 (3.0-3)	92.52	91.33	87.19	96.23	95.53	89.58	100.00	96.94	89.99	97.18	95.85	89.77
E8 (3.0-7)	91.65	92.20	86.54	94.91	96.11	90.02	95.75	100.00	90.72	96.47	96.62	90.43
E9 (3.0-15)	86.41	87.59	90.94	89.43	90.95	95.69	90.65	91.14	100.00	89.46	90.35	97.31
E10 (3.5-3)	91.61	90.45	85.21	95.80	94.21	88.95	96.10	96.37	90.07	100.00	96.71	90.06
E11 (3.5-7)	91.00	91.12	86.78	94.77	95.48	89.99	97.27	95.91	91.01	96.59	100.00	91.18
E12 (3.5-15)	86.90	86.05	90.54	89.22	89.44	94.67	89.96	90.42	95.54	90.54	91.43	100.00

(1) For each dataset, the criteria considered (in parentheses) were: standard deviations around the mean for outlier removal, and minimum number of animals per contemporary group, respectively.

Table S6. Concordance in EBV ranking across the evaluated scenarios for early pregnancy (EP) in Nellore heifers. The upper diagonal presents the percentage of common animals among the top 1% of males and top 20% of females, whereas the lower diagonal presents the percentage concordance in the ranking of all animals.

Datasets[(1)]	Datasets[(1)]					
	E1	E2	E3	E4	E5	E6
E1 (CV-3)	100	96.66	97.75	96.96	97.27	96.88
E2 (SV-3)	94.32	100	96.63	97.53	96.18	96.82
E3 (CV-7)	94.41	94.23	100	97.01	97.32	96.97
E4 (SV-7)	94.31	94.41	94.28	100	96.52	96.97
E5 (CV-15)	94.07	93.85	94.08	93.95	100	97.34
E6 (CV-15)	94.04	93.95	94.05	94.00	94.27	100

(1) For each dataset, the criteria considered (in parentheses) were: exclusion with and without variability, and minimum number of animals per contemporary group, respectively.

Table S7. Concordance in EBV ranking across the evaluated scenarios for number of calvings at 53 months of age (NC53) in Nellore females. The upper diagonal presents the percentage of common animals among the top 1% of males and top 20% of females, whereas the lower diagonal presents the percentage concordance in the ranking of all animals.

Datasets[(1)]	Datasets[(1)]					
	E1	E2	E3	E4	E5	E6
E1 (CV-3)	100.00	98.59	97.95	98.64	98.60	97.94
E2 (SV-3)	98.79	100.00	97.94	98.51	98.65	97.93
E3 (CV-7)	98.48	98.51	100.00	97.85	97.94	98.64
E4 (SV-7)	98.82	98.80	98.48	100.00	98.58	97.87
E5 (CV-15)	98.79	98.82	98.52	98.79	100.00	97.95
E6 (SV-15)	98.48	98.51	98.79	98.49	98.52	100.00

(1) For each dataset, the criteria considered (in parentheses) were: exclusion with and without variability, and minimum number of animals per contemporary group, respectively.

Table S8. Concordance in EBV ranking across the evaluated scenarios for scrotal circumference at 18 months (SC18) in Nellore males. The upper diagonal presents the percentage of common animals among the top 1% of males and top 20% of females, whereas the lower diagonal presents the percentage concordance in the ranking of all animals.

Datasets[(1)]	Datasets[(1)]											
	E1	E2	E3	E4	E5	E6	E7	E8	E9	E10	E11	E12
E1 (2.0-3)	100.00	100.00	94.11	90.37	90.37	87.65	88.23	88.23	85.86	87.71	87.71	85.38
E2 (2.0-7)	100.00	100.00	94.11	90.37	90.37	87.65	88.23	88.23	85.86	87.71	87.71	85.38
E3 (2.0-15)	97.59	97.59	100.00	87.00	87.00	90.38	85.13	85.13	88.24	84.7	84.7	87.7
E4 (2.5-3)	96.72	96.72	94.66	100.00	100.00	94.26	95.74	95.74	92.07	94.97	94.97	91.45
E5 (2.5-7)	96.72	96.72	94.66	100.00	100.00	94.26	95.74	95.74	92.07	94.97	94.97	91.45
E6 (2.5-15)	94.84	94.84	96.69	97.59	94.59	100.00	91.60	91.6	95.74	91.03	91.03	94.98
E7 (3.0-3)	95.78	95.78	93.77	98.96	98.96	96.64	100.00	100	94.37	98.53	98.53	93.81
E8 (3.0-7)	95.78	95.78	93.77	98.96	98.96	96.64	100.00	100.00	94.37	98.53	98.53	93.81
E9 (3.0-15)	93.94	93.94	95.74	96.64	96.64	98.93	97.64	97.64	100.00	93.68	93.68	98.45
E10 (3.5-3)	95.53	95.53	93.55	98.68	98.68	96.40	99.70	99.7	97.4	100.00	100	94.43
E11 (3.5-7)	95.53	95.53	93.55	98.68	98.68	96.40	99.70	99.7	97.4	100	100.00	94.43
E12 (3.5-15)	93.68	93.68	95.47	96.36	96.36	98.63	97.36	97.36	99.68	97.67	97.67	100.00

[(1)] For each dataset, the criteria considered (in parentheses) were: standard deviations around the mean for outlier removal, and minimum number of animals per contemporary group, respectively.

Table S9. Number of genetic links, mean percentage of animals (AP), and connected contemporary groups (CCc) for the evaluated models (MD1–8) for age at first calving (AFC).

**MD**	**MD1**	**MD2**	**MD3**	**MD4**	**MD5**	**MD6**	**MD7**	**MD8**	$\underline{x}$ **(se)**
				**MCGS 3**					
**MLG**	10362.26	7452.66	20805.48	6942.02	13868.34	6942.01	4410.91	5631.77	10416.77
SD_{MLG}	296.74	221.87	905.73	196.15	576.83	196.15	100.31	205.22	(1402.98)
**AP**	98.80%	97.95%	99.16%	97.85%	98.59%	97.85%	97.22%	95.63%	98.06%
SD_{AP}	0.04	0.05	0.02	0.06	0.06	0.06	0.07	0.14	(0.27)
**CGc**	94.77%	93.31%	95.95%	93.36%	94.47%	93.36%	92.92%	91.46%	93.87%
SD_{GCc}	0.11	0.08	0.09	0.07	0.17	0.07	0.08	0.18	(0.33)
				**MCGS 7**					
**MLG**	12090.73	8795.02	21983.19	8302.27	15079.18	8302.27	5325.75	6151.31	11632.87
SD_{MLG}	357.74	272.53	961.48	246.46	643.52	246.46	135.05	225.72	(1420.36)
**AP**	99.27%	98.57%	99.24%	97.73%	98.79%	98.48%	98.29%	96.38%	98.43%
SD_{AP}	0.15	0.03	0.02	0.04	0.05	0.04	0.06	0.15	(0.22)
**CGc**	97.83%	96.90%	97.33%	96.72%	96.50%	96.72%	96.71%	94.35%	96.65%
SD_{GCc}	0.08	0.08	0.08	0.08	0.09	0.08	0.10	0.21	(0.24)
				**MCGS 15**					
**MLG**	13956.09	10155.87	24300.45	9601.54	17231.47	9601.54	5780.18	6731.13	13221.62
SD_{MLG}	422.29	322.91	1060.66	291.65	739.72	291.65	155.25	245.11	(1590.34)
**AP**	99.43%	98.93%	99.38%	98.91%	99.09%	98.91%	99.26%	97.25%	98.93%

SD_{AP}	0.01	0.03	0.02	0.03	0.05	0.03	0.05	0.14	(0.16)
**CGc**	98.84%	98.09%	98.36%	98.06%	98.10%	98.06%	98.89%	96.34%	98.12%
SD_{GCc}	0.02	0.06	0.09	0.07	0.07	0.07	0.09	0.16	(0.18)

MDi: models evaluated where i = 1, 2, ..., 8; TMGC: minimum size per contemporary group; MLG: mean genetic linkage; $SD_{MLG:}$ standard deviation per model for the mean genetic linkage; AP: mean percentage of connected animals; $SD_{AP:}$ standard deviation per model for the percentage of connected animals; GCc: average percentage of connected groups; SD_{GCc}: standard deviation per model for the percentage of connected groups; : overall mean by size; (se): standard error for the mean per model of genetic links, percentage of connected animals, and connected groups.

Table S10. Number of genetic links, mean percentage of animals (AP), and connected contemporary groups (CG) for the evaluated models (MD1–5) for early pregnancy (EP).

**MD**	**MD1**	**MD2**	**MD3**	**MD4**	**MD5**	x **(se)**
			**MCGS 3**			
**MLG**	34550.21	34705.14	54503.97	4742.14	12499.18	28200.13
SD_{MLG}	5882.25	5895.51	7319.11	3312.01	4514.92	(5384.76)
**AP**	99.31%	99.33%	99.65%	97.83%	97.77%	98.78%
SD_{AP}	0.0008	0.0009	0.0018	0.0007	0.0021	(0.00)
**CGc**	97.95%	97.99%	98.88%	93.75%	96.08%	0.97%
SD_{GCc}	0.0103	0.0105	0.0066	0.0054	0.0004	(0.01)
			**MCGS 7**			
**MLG**	35287.47	35449.25	54612.56	5558.66	12577.25	28697.04
SD_{MLG}	6577.76	6597.73	7432.39	4134.79	4569.44	(5862.420
**AP**	99.32%	99.35%	99.65%	98.28%	97.78%	99%
SD_{AP}	0.0006	0.0007	0.0018	0.0031	0.0021	(0.00)
**CGc**	98.30%	98.34%	98.87%	96.44%	96.16%	98%
SD_{GCc}	0.0075	0.0076	0.0066	0.0048	0.0004	(0.01)
			**MCGS 15**			
**MLG**	37750.05	37902.8	56678.44	6450.14	13601.13	30476.51
SD_{MLG}	8029.47	8061.94	8904.14	4763.74	4923.93	(6936.64)
**AP**	99.38%	99.40%	99.67%	98.72%	98.06%	99%
SD_{AP}	0.0001	0.0001	0.0016	0.0028	0.0028	(0.00)
**CGc**	98.72%	98.75%	99.06%	98.03%	97.03%	98%

SD_{GCc}	0.0037	0.004	0.0058	0.0008	0.0025	(0.00)

MDi: modelos avaliados em que i = 1, 2 ...5; TMGC: tamanho mínimo por grupo de contemporâneo; MLG: média de laços genéticos; SD_{MLG}: desvio-padrão por modelo para a média de laços genéticos; AP: porcentagem média de animais conectados; SD_{AP}: desvio-padrão por modelo referente a porcentagem de animais conectados; GCc: porcentagem média de grupos conectados; SD_{GCc}: desvio-padrão por modelo referente a porcentagem de grupos conectados; : média geral por tamanho; (se): erro padrão referente a média por modelo dos laços genéticos, porcentagem de animais e grupos conectados.

Table S11. Number of genetic links, mean percentage of animals (AP), and connected contemporary groups (CGc) for the evaluated models (MD1–4) for the number of calvings by 53 months of age.

MD	MD1	MD2	MD3	MD4	x (se)
			**MCGS 3**		
**MLG**	32256.06	32388.87	49513.58	32256.06	36603.64
SD_{MLG}	308.01	310.72	12.49	308.01	(5755.51)
**AP**	99.19%	99.21%	99.47%	99.19%	99.26%
SD_{AP}	0.00021	0.00021	0.000	0.000	(0.38)
**CGc**	97.00%	97.03%	98.21%	97.00%	97.31%
SD_{GCc}	0.0019	0.0017	0.0000	0.0019	(0.40)
			**MCGS 7**		
**MLG**	33023.17	33162.93	49513.58	33023.17	37180.71
SD_{MLG}	180.17	181.83	12.49	786.52	(5766.22)
**AP**	99.21%	99.23%	99.47%	99.21%	99.28%
SD_{AP}	0.00014	0.00014	0.000	0.000	(0.39)
**CGc**	97.52%	97.55%	98.21%	97.52%	97.70%
SD_{GCc}	0.0000	0.00071	0.0000	0.0000	(0.42)
			**MCGS 15**		
**MLG**	35758.70	35904.21	51912.19	14120.87	34423.99
SD_{MLG}	70.35	70.35	0.0000	70.35	(7251.90)
**AP**	99.31%	99.33%	99.50%	99.31%	99.36%
SD_{AP}	0.00014	0.000	0.000	0.000	(0.36)
**CGc**	98.28%	98.30%	98.53%	98.28%	98.35%

SD_{GCc}	0.00014	0.0000	0.000	0.00014	(0.40)

MDi: evaluated models where i = 1, 2, ..., 4; TMGC: minimum size per contemporary group; MLG: mean genetic linkage; SD_{MLG}: standard deviation per model for the mean genetic linkage; AP: mean percentage of connected animals; SD_{AP}: standard deviation per model for the percentage of connected animals; GCc: average percentage of connected groups; SD_{GCc}: standard deviation per model for the percentage of connected groups; : overall mean by size; (se): standard error for the mean per model of genetic links, percentage of connected animals, and connected groups.

Table S12. Number of genetic links, mean percentage of animals (AP), and connected contemporary groups (CGc) for the evaluated models (MD1–7) for scrotal circumference (SC18).

MD	MD1	MD2	MD3	MD4	MD5	MD6	MD7	$\underline{x}$ (se)
				**MCGS3**				
**MLG**	117150.785	117812.2	175632.6	117150.8	51256.13	117812.2	175633	124635.33
SD_{MLG}	27417.54	27527.83	38519.62	27417.54	12839.24	27527.83	38519.62	(16029.06)
**AP**	94.38%	94.51%	96.92%	94.38%	91.46%	94.45%	96.92%	94.71%
SD_{AP}	0.09	0.18	0.08	0.09	0.13	0.09	0.08	(0.65)
**CGc**	97.81%	97.84%	98.89%	97.81%	94.01%	97.84%	98.89%	97.58%
SD_{GCC}	0.05	0.05	0.03	0.05	0.13	0.05	0.03	(0.62)
				**MCGS7**				
**MLG**	120033.53	120731.8	175632.6	120033.5	51256.13	120731.78	175633	126293.133
SD_{MLG}	28089.99	28207.66	38519.62	28089.99	12839.24	28207.66	38519.62	(15916.13)
**AP**	95.15%	95.21%	96.92%	95.15%	91.46%	95.21%	96.92%	95.14%
SD_{AP}	0.09	0.09	0.08	0.09	0.13	0.09	0.08	(0.70)
**CGc**	97.86%	97.88%	98.89%	97.86%	94.015%	97.88%	98.89%	97.61%
SD_{GCC}	0.05	0.05	0.03	0.05	0.13	0.05	0.03	(0.62)
				**MCGS15**				
**MLG**	130982.023	131646.7	185109.4	130982	54829.57	131646.73	185109	135757.967
SD_{MLG}	30730.74	30836.45	40723.85	30730.74	13912.00	30836.45	40723.85	(16547.15)
**AP**	96.47%	96.47%	97.77%	96.47%	93.14%	96.47%	97.77%	96.37%
SD_{AP}	0.06	0.06	0.07	0.06	0.13	0.06	0.07	(0.60)
**CGc**	98.04%	98.05%	98.98%	98.04%	94.65%	98.05%	98.98%	97.83%
SD_{GCC}	0.04	0.04	0.03	0.04	0.14	0.04	0.03	(0.55)

MDi: evaluated models where i = 1, 2, ..., 7; MCGS: minimum size per contemporary group; MLG: mean genetic linkage; SD_{MLG}: standard deviation per model for the mean genetic linkage; AP: mean percentage of connected animals; SD_{AP}: standard deviation per model for the percentage of connected animals; GCc: average percentage of connected groups; SD_{GCc}: standard deviation per model for the percentage of connected groups; : overall mean by size; (se): standard error for the mean per model of genetic links, percentage of connected animals, and connected groups.
